# Full-Genome Sequence of Pantropic Canine Coronavirus

**DOI:** 10.1128/genomeA.00401-15

**Published:** 2015-05-07

**Authors:** Nicola Decaro, Viviana Mari, Giulia Dowgier, Gabriella Elia, Gianvito Lanave, Maria Loredana Colaianni, Canio Buonavoglia

**Affiliations:** Department of Veterinary Medicine, University of Bari, Valenzano, Italy

## Abstract

Pantropic canine coronavirus (CCoV) was first detected in young dogs in Italy in 2005, but the complete genome sequence of this virus had not yet been determined. Here, we report the full-length genome sequence of the prototype strain CB/05, which showed that this virus is genetically similar to CCoV-IIa viruses.

## GENOME ANNOUNCEMENT

Canine coronavirus (CCoV), a member of the *Alphacoronavirus* genus of the *Coronaviridae* family, is an enveloped, positive-sense, single-strand RNA virus (1, 2). Two distinct genotypes have been reported, namely, CCoV-I and CCoV-II, with CCoV-II being classified into two subgenotypes, CCoV-IIa and CCoV-IIb, on the basis of the genetic relatedness to transmissible gastroenteritis virus (TGEV) of swine (1). Although the virus is generally responsible for mild gastroenteritis, a CCoV-IIa pantropic variant, strain CB/05, was associated with systemic fatal disease in young dogs (3), and similar virulent strains were detected in several countries (4–6). To date, only the 3′ end of the CB/05 genome is publicly available (7). Thus, the aim of the present study was to determine the full-length genome of this pantropic CCoV.

Viral RNA was extracted from the original lung sample using the QIAamp viral RNA mini kit (Qiagen), and overlapping reverse transcription-PCR (RT-PCR) assays were carried out using primers designed in alphacoronavirus conserved regions. The very 5′ and 3′ ends were amplified using the Rapid Amplification of cDNA Ends (RACE) system (Invitrogen). The RT-PCR products were subjected to direct sequencing, and the obtained sequences were assembled and analyzed using the Geneious platform.

The CB/05 genome is 29,266 nucleotide (nt) long, excluding the 3′ poly(A) tail, and shows the typical alphacoronavirus-1 organization: a 5′ untranslated region (UTR) (nucleotides [nt] 1 to 313), replicase complex (open reading frame 1ab [ORF1ab], nt 314 to 20358), spike (S) gene (nt 20355 to 24719), membrane (M) gene (nt 26086 to 26874), nucleocapsid (N) gene (nt 26887 to 28035), and a 3′ UTR (nt 28992 to 29266). Two regions encoding accessory proteins were detected in the genome, ORF3abc (nt 24,772 to 25,840), between the S and E genes, and ORF7ab (nt 28040 to 28991), downstream of the N gene. These gene groups exhibited the same organization as that in other CCoVs, with the exception of ORF1b, whose product was 22 amino acids (aa) long (49 amino acids shorter than expected) due to the presence of a 38-nt deletion and to a frameshift in the sequence downstream of the deletion that introduced an early stop codon. As in other CCoV-II strains, only remnants of ORF3 were evident in the CB/05 genome, while the CCoV-I genome had been found to harbor the entire coding sequence. Transcription regulatory sequences preceding each of the 7 putative mRNAs encoding the structural and nonstructural proteins contained the conserved core CUAAAC.

Alignment of the complete genome sequences of strain CB/05 and reference alphacoronaviruses showed the closest genetic relatedness with CCoV-IIa isolates (92.07 to 93.49% nt identity), followed by TGEV (90.31%) and porcine respiratory coronavirus (87.67%). CCoV-I and feline coronaviruses (FCoVs) were more distantly related (nt identities, 84.16% and 77.25 to 83.81%, respectively). No comparison was possible with CCoV-IIb, since there are no full-length genome sequences available in the GenBank database for these viruses.

In conclusion, strain CB/05 is genetically similar to other CCoV-IIa isolates, with the exception of the ORF3b deletion. To what extent this deletion is associated with virus pathogenicity is currently unknown, since it was not detected in other recently reported pantropic strains (6).

### Nucleotide sequence accession number.

The complete genome sequence of CCoV-IIa strain CB/05 was deposited at GenBank under the accession no. KP981644.
